# Rapid Diagnosis of Group A Streptococcal Septic Arthritis of the Knees in the Emergency Department Using Point-of-Care Gram Staining of the Synovial Fluid

**DOI:** 10.7759/cureus.91394

**Published:** 2025-09-01

**Authors:** Tomohiro Taniguchi, Hayato Tatsumi, Yasumitsu Fujii, Sonoko Miyoshi

**Affiliations:** 1 General Internal Medicine and Infectious Diseases, Hiroshima Prefectural Hospital, Hiroshima, JPN; 2 Orthopaedic Surgery, Hiroshima Prefectural Hospital, Hiroshima, JPN

**Keywords:** group a streptococcus, point-of-care gram staining, septic arthritis, streptococcus pyogenes, synovial fluid

## Abstract

Group A *Streptococcus* (*Streptococcus pyogenes*) infections are increasing globally and can be invasive despite immunocompetence. Septic arthritis requires an early diagnosis and prompt treatment. Because of its high specificity, Gram staining of the synovial fluid is crucial to early diagnoses; however, its sensitivity is low, and around-the-clock laboratory testing is often impractical. We report a case of oligoarticular septic arthritis in an immunocompetent man in his 60s. Point-of-care Gram staining of the synovial fluid revealed chains of Gram-positive cocci. An invasive streptococcal infection was suspected. Vancomycin was administered within 30 minutes of the left knee aspiration, as the patient was considered to be at increased risk for methicillin-resistant *Staphylococcus aureus* infection. Ceftriaxone was subsequently initiated after both knees were aspirated by the orthopedic surgeon, since beta-lactams are generally more effective than vancomycin against *Streptococcus*. After *S. pyogenes* was isolated from the blood and synovial fluid cultures, antimicrobial therapy was narrowed to penicillin G and clindamycin and was subsequently changed to ampicillin. Early diagnosis and treatment can improve the prognosis of such cases. Point-of-care Gram staining of the synovial fluid can play a crucial role in antimicrobial stewardship and improve the outcomes of patients with septic arthritis.

## Introduction

The incidence of group A *Streptococcus* (GAS) infections has increased globally since the 1980s, with a particularly notable rise following the coronavirus disease 2019 (COVID-19) pandemic [1,2]. GAS infections can lead to a broad spectrum of clinical manifestations that can progress to invasive diseases, even in immunocompetent individuals [3]. GAS-induced septic arthritis is a serious condition in which rapid diagnosis and prompt initiation of treatment are essential for optimal outcomes. Although a positive synovial fluid culture is considered the gold standard for diagnosing septic arthritis, it typically requires 2-3 days to yield results, potentially delaying treatment and increasing the risk of joint destruction [4]. Although rapid antigen detection tests are widely used for the diagnosis of GAS pharyngitis, their application to synovial fluid specimens is uncommon and not routinely recommended. Therefore, clinicians must rely on more rapidly available laboratory tests, such as synovial fluid white blood cell counts, to make a timely diagnosis [4]. Gram staining of the synovial fluid is a traditional diagnostic examination that provides rapid results and facilitates early diagnoses; however, its sensitivity is relatively low [5]. Moreover, Gram staining is typically performed by technicians in laboratory settings; however, 24-hour availability of trained personnel is challenging and impractical. In Japan, Gram staining is regarded as a fundamental clinical skill that residents are expected to acquire during their training [6]. Physicians are permitted to perform point-of-care Gram staining under the supervision of attending physicians to diagnose respiratory, urinary tract, and central nervous system infections [7-11]. We report a case of invasive GAS-induced septic arthritis of the knees that was diagnosed early using point-of-care Gram staining performed by a physician. This technique enabled the initiation of targeted therapy with vancomycin and ceftriaxone within 30 minutes of synovial fluid aspiration, without the need for broad-spectrum agents against *Pseudomonas aeruginosa* or drug-resistant *Enterobacterales*.

## Case presentation

A man in his sixth decade of life presented to the emergency department on a Sunday morning because of a three-day history of left knee pain and a two-day history of right knee pain that caused significant difficulty with walking. An infectious disease specialist was on duty in the emergency department that day. Additional symptoms included decreased appetite and urinary incontinence. Although the patient was initially afebrile, a fever of 37.7°C was subsequently recorded. Immediately before presentation, the patient experienced severe bilateral knee pain and was unable to move. Subsequently, an ambulance transported him to the emergency department of the internal medicine unit. The patient also reported mild bilateral shoulder pain that was initially considered attributable to compensatory overuse as a result of restricted knee mobility.

The patient's medical history was notable for type A aortic dissection and artificial graft replacement two years prior to presentation, as well as hypertension and a right meniscus injury sustained 17 years previously that had been treated with a bolt (the bolt was later removed). The patient had no prior history of methicillin-resistant *Staphylococcus aureus* (MRSA) detection. He also had no known allergies. Additionally, the patient was using bisoprolol, nifedipine, and perindopril, resided with his wife and daughter, and had not been exposed to individuals with infectious diseases. He denied any recent sexual behaviors considered a risk for sexually transmitted infections.

**Figure 1:**
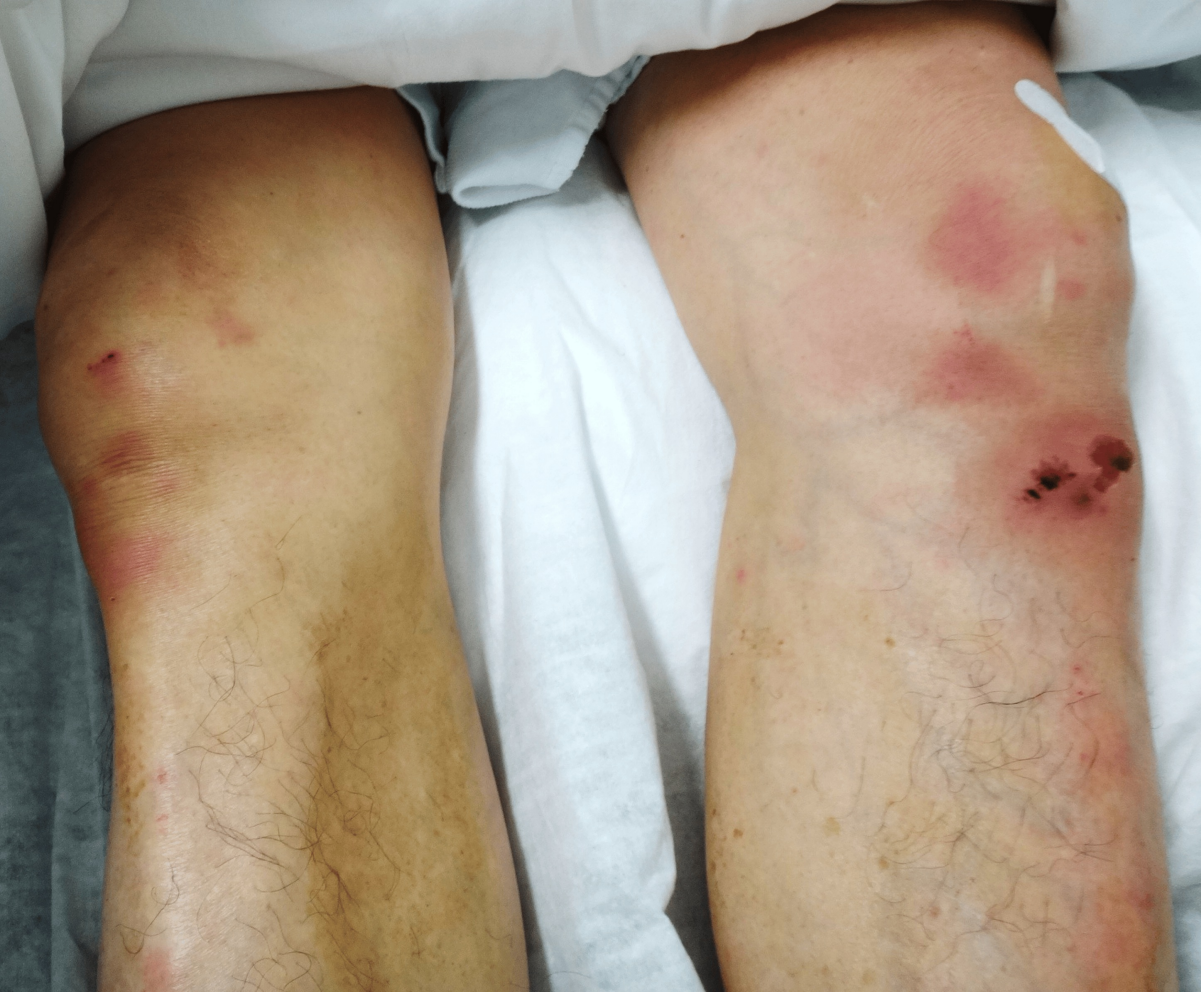
Bilateral knee swelling after left knee arthrocentesis

At the time of presentation, his vital signs were as follows: blood pressure, 136/92 mmHg; heart rate, 98 beats per minute; respiratory rate, 20 breaths per minute; body temperature, 38.7°C; and peripheral oxygen saturation, 96% on ambient air. He has a height of 172 cm, a weight of 65 kg, and a BMI of 21.9. The patient appeared generally unwell but was conscious and lucid. A physical examination revealed no signs of anemia or jaundice; however, the oral cavity was dry and poorly maintained. Cardiac auscultation revealed normal heart sounds without murmurs. The chest was clear to auscultation and exhibited surgical scars from prior interventions. The abdominal examination was unremarkable, and tenderness was not observed. The shoulder joints did not exhibit swelling, paresthesia, or skin abnormalities. No significant lymphadenopathy or signs of lymphangitis were observed in the inguinal or femoral regions. Both knees were swollen, but swelling was more pronounced on the left side than on the right side (Figure 1). Localized erythema was noted on the skin over both knees, along with abrasions and crusting, which were considered to be pressure-related changes secondary to limited mobility. The patient denied any history of knee trauma prior to symptom onset.

On palpation of the lower extremities, both knees exhibited warmth and swelling. Passive and active movement elicited moderate pain in the right knee and severe pain in the left knee. The patient had difficulty moving from a supine position and was unable to bear weight on either knee. 

Table 1 shows the results of the blood tests. The patient's serum creatinine level was 0.7 mg/dL one year before admission. 

**Table 1:** Blood tests

Laboratory test	Result	Reference range
White blood cell count (/μL)	7,800	4,000-10,000
Hemoglobin level (g/dL)	15.2	13.5-17.5
Platelet count (/μL)	26,000	150,000-450,000
Sodium (mmol/L)	125	135-145
Potassium (mmol/L)	3.7	3.6-4.8
Creatine kinase (IU/L)	38,318	38-174
Aspartate aminotransferase (IU/L)	548	10-40
Alanine aminotransferase (IU/L)	150	7-56
Lactate dehydrogenase (IU/L)	1,330	140-280
Total bilirubin (mg/dL)	3.2	0.2-1.2
Blood urea nitrogen (mg/dL)	70.6	7-20
Creatinine (mg/dL)	3.6	0.7-1.3
C-reactive protein (mg/dL)	39.5	<0.3

Nasopharyngeal antigen test results yielded negative results for influenza and severe acute respiratory syndrome coronavirus 2 (SARS-CoV-2).

**Figure 2:**
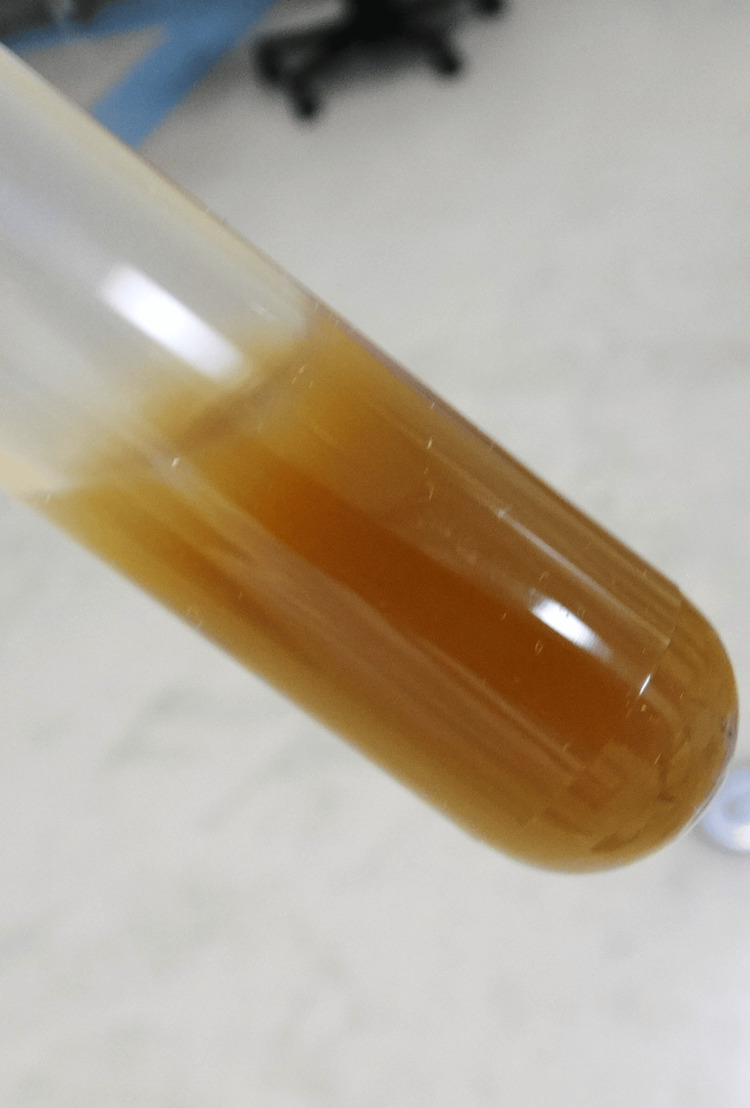
Brown synovial fluid

Echocardiography and non-contrast computed tomography confirmed effusion in both knee joints; however, no obvious alternative infectious focus was identified, including in the area of the prior aortic graft. Transthoracic echocardiography revealed neither valvular disease nor vegetations. Arthrocentesis of the left knee yielded 20 mL of brown synovial fluid (Figure 2). The synovial fluid was viscous and had no foul odor. At this hospital, the devices used to measure synovial fluid white blood cell counts and biochemical results are not operational on weekends; therefore, the white blood cell counts could not be obtained.

**Figure 3:**
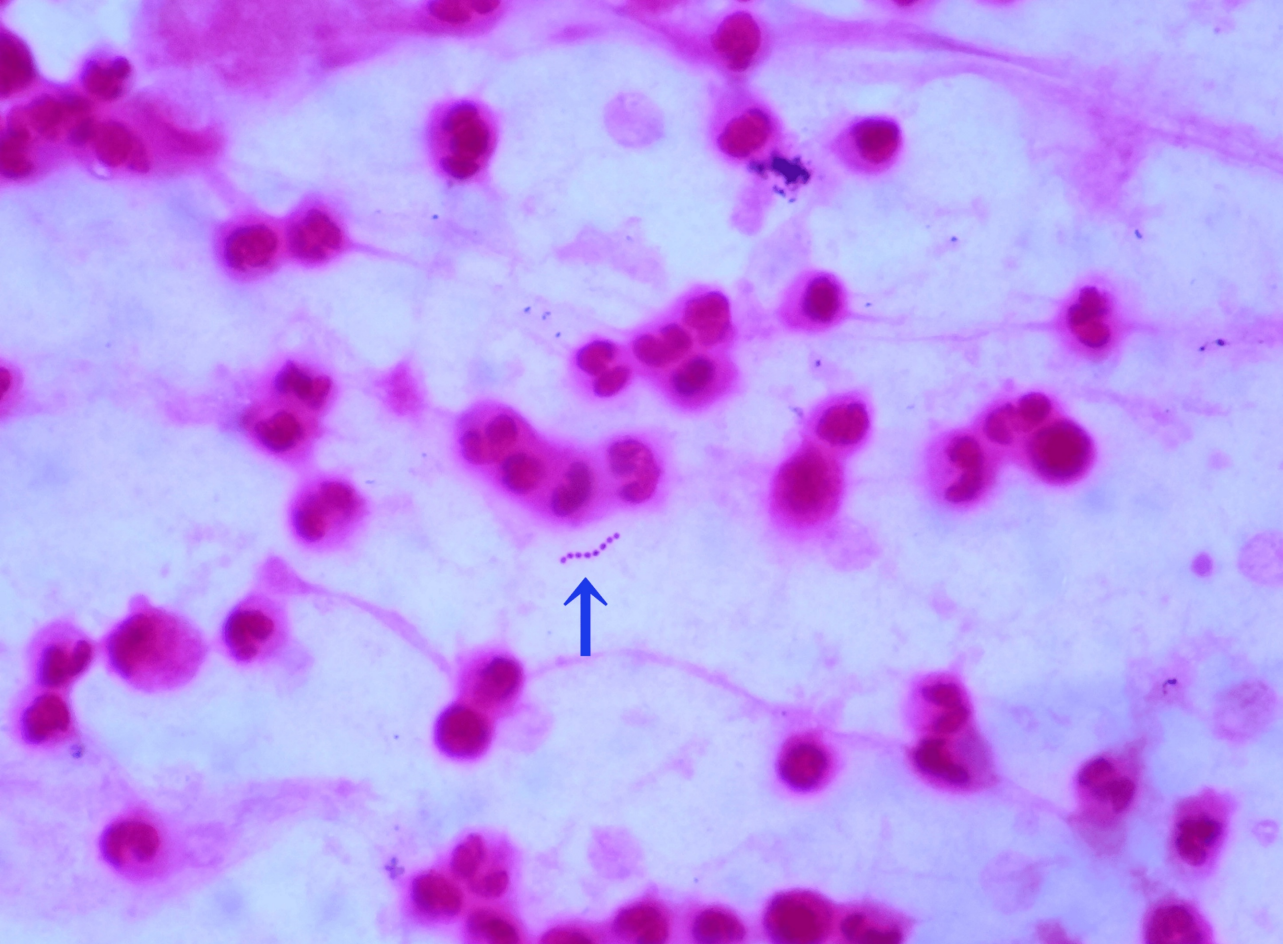
Gram-positive cocci in chains observed extracellularly on point-of-care Gram staining of the synovial fluid Numerous polymorphonuclear leukocytes were observed. Extracellular Gram-positive cocci chains (arrow) are confirmed.

**Figure 4:**
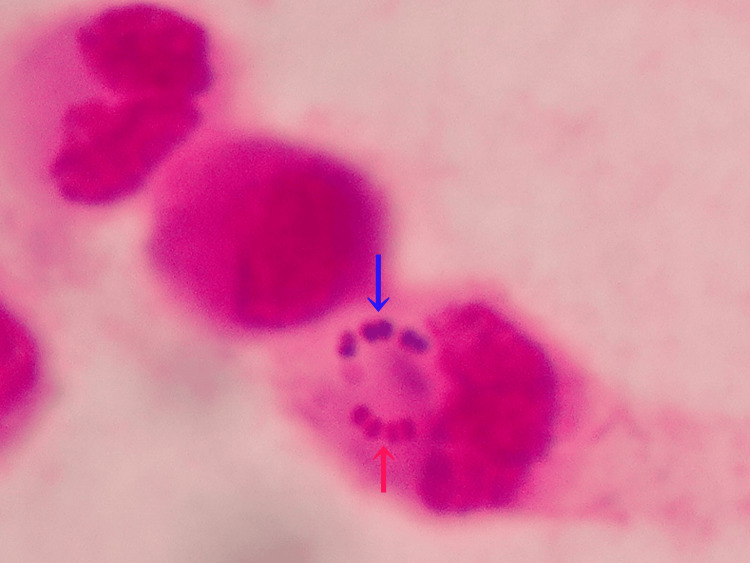
Gram-positive cocci phagocytosed by leukocytes on point-of-care Gram staining of the synovial fluid Gram-positive cocci in chains (blue arrow) and Gram-negative cocci in chains (red arrow) phagocytosed by leukocytes.

Point-of-care Gram staining of the uncentrifuged synovial fluid was performed by a postgraduate year 2 resident under the guidance of an infectious disease specialist. The results indicated abundant neutrophils (Figure 3) containing phagocytosed Gram-positive and Gram-negative cocci (Figure 4) as well as occasional chains of extracellular Gram-positive cocci (Figure 3).

Methylene blue staining was not performed. Uric acid and calcium pyrophosphate crystals were not detected. 

The patient was diagnosed with septic arthritis of the knees caused by Gram-positive cocci. Gram staining findings suggested that a streptococcal infection was the most likely etiology rather than *Staphylococcus aureus* or *Neisseria gonorrhoeae*. However, given the possibility of Gram-negative cocci, *N. gonorrhoeae* could not be ruled out. Synovial fluid samples and two sets of blood cultures were obtained.

Because the patient had undergone cardiac surgery two years earlier, MRSA colonization was considered a potential risk. Vancomycin was administered within 30 minutes of the left knee aspiration. Because *N. gonorrhoeae* can cause polyarthritis and vancomycin is ineffective against this pathogen, ceftriaxone, to target streptococci and gonococci, was subsequently initiated after both knees were aspirated by the orthopedic surgeon, since beta-lactams are generally more effective than vancomycin against *Streptococcus*. Surgery of the knees was planned; however, despite aggressive rehydration with extracellular fluids, the patient developed shock after 12 hours on arrival and was admitted to the intensive care unit, where catecholamine support and continuous hemodiafiltration (CHDF) were initiated. Intermittent joint punctures were performed, and in addition, drainage tubes were placed with repeated washouts in both knees. The initial two sets of blood cultures and the synovial fluid culture confirmed GAS. As the patient fulfilled the criteria of shock requiring catecholamines, renal impairment, coagulopathy, and liver involvement, a diagnosis of bilateral knee septic arthritis complicated by septic shock and toxic shock syndrome was established. The third set of blood cultures, obtained after the initiation of vancomycin and ceftriaxone, was negative. The subsequent onset of left shoulder pain and swelling necessitated arthrocentesis of the left shoulder, which yielded 10 mL of purulent synovial fluid; nevertheless, no organisms were isolated on culture.

There was no apparent trauma, and symptoms began with left knee pain, followed by right knee involvement the next day. The joint pain was severe, leading to immobility and urinary incontinence. Physical examination showed clear signs of septic arthritis with minimal skin inflammation. No cardiac murmurs were detected, and transthoracic echocardiography showed no valvular disease or vegetations. Additionally, there were no signs of infection around the aortic graft, and blood cultures rapidly turned negative after the initiation of antimicrobial therapy. The patient had a history of right knee surgery. Although the portal of entry for GAS was unclear, the clinical course suggests hematogenous spread, initially to the left knee, then to the previously operated right knee, and later to the left shoulder.

After the confirmation of GAS susceptibility, antimicrobial therapy was changed to penicillin G (4 million units every six hours; minimum inhibitory concentration <0.03 μg/mL) and clindamycin (600 mg every six hours). Renal function and serum potassium were monitored daily in the intensive care unit. During CHDF, serum creatinine remained between 2.7 and 3.6 mg/dL and potassium between 3.4 and 4.6 mEq/L; thus, hyperkalemia associated with penicillin G administration did not become an issue. Fluid removal was maintained at 1200-1300 mL/day, with urine output of approximately 500 mL/day. CHDF was discontinued after four days, and urine output subsequently increased to 1000-1500 mL/day. At the time of discontinuation, penicillin G was replaced with ampicillin (1 g every six hours) in order to reduce the risk of potassium burden. Clindamycin was discontinued after four days, as hemodynamic stability was achieved, renal function deterioration had ceased, and urine output was maintained. The serum creatinine level was 3.6 mg/dL at this point.

After the patient's condition stabilized and inflammatory marker levels declined, surgical drainage of the left shoulder and both knees was performed in the operating room on day 13 of hospitalization. Upon incision of the joint capsules of both knees, hematoma and serous synovial fluid were observed. The hematoma and damaged synovium were debrided, the joint was irrigated, and the wound was closed. In the left shoulder joint, partial rupture of the supraspinatus and rotator cuff injury were identified. A drainage tube was inserted into the joint space, and serous fluid was drained. The joint was irrigated, a drain was placed, and the wound was closed by suturing. The synovial fluid cultures obtained during surgery were also negative. Rehabilitation was initiated to improve the joint range of motion.

**Figure 5:**
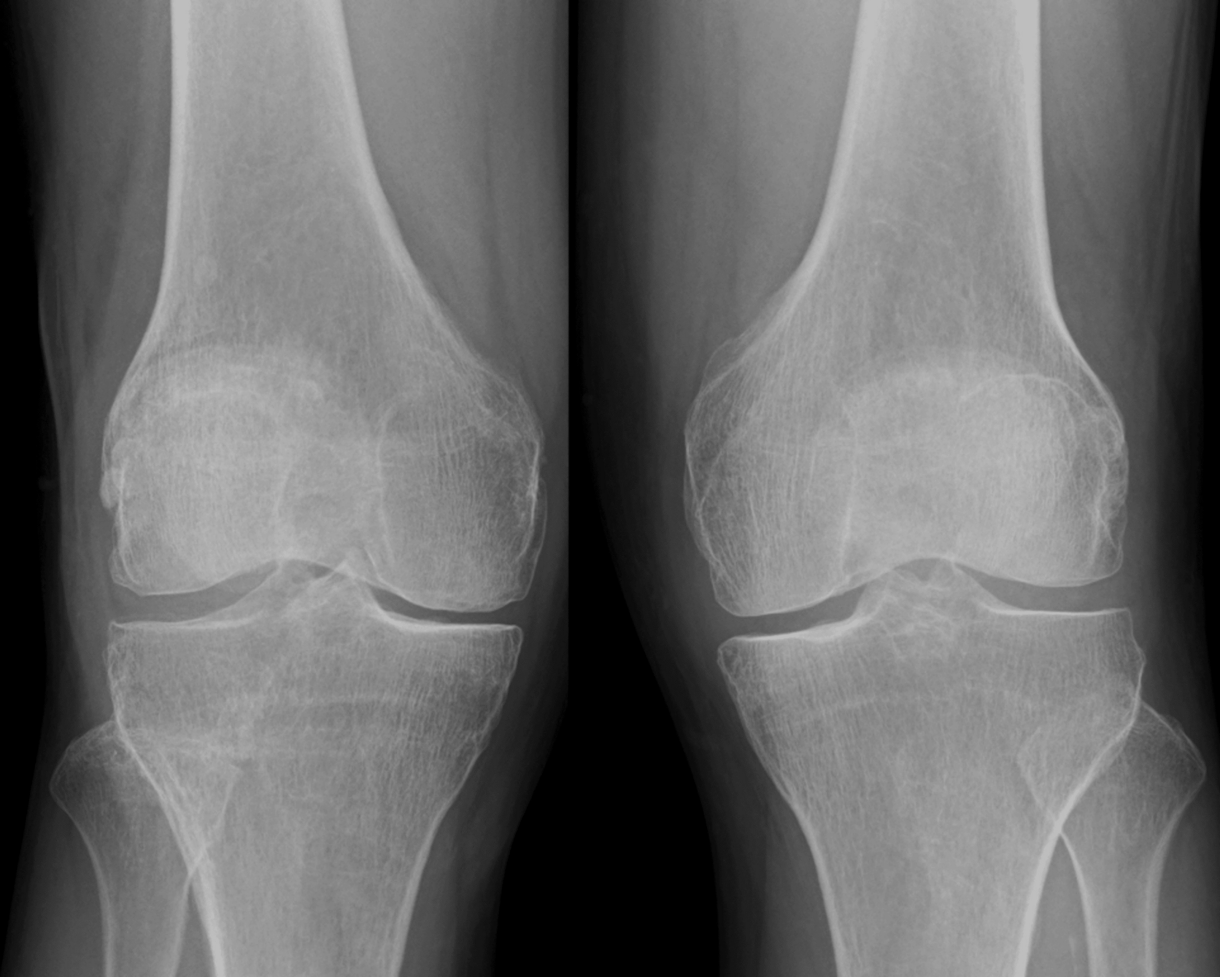
Bilateral knee X-rays on admission

**Figure 6:**
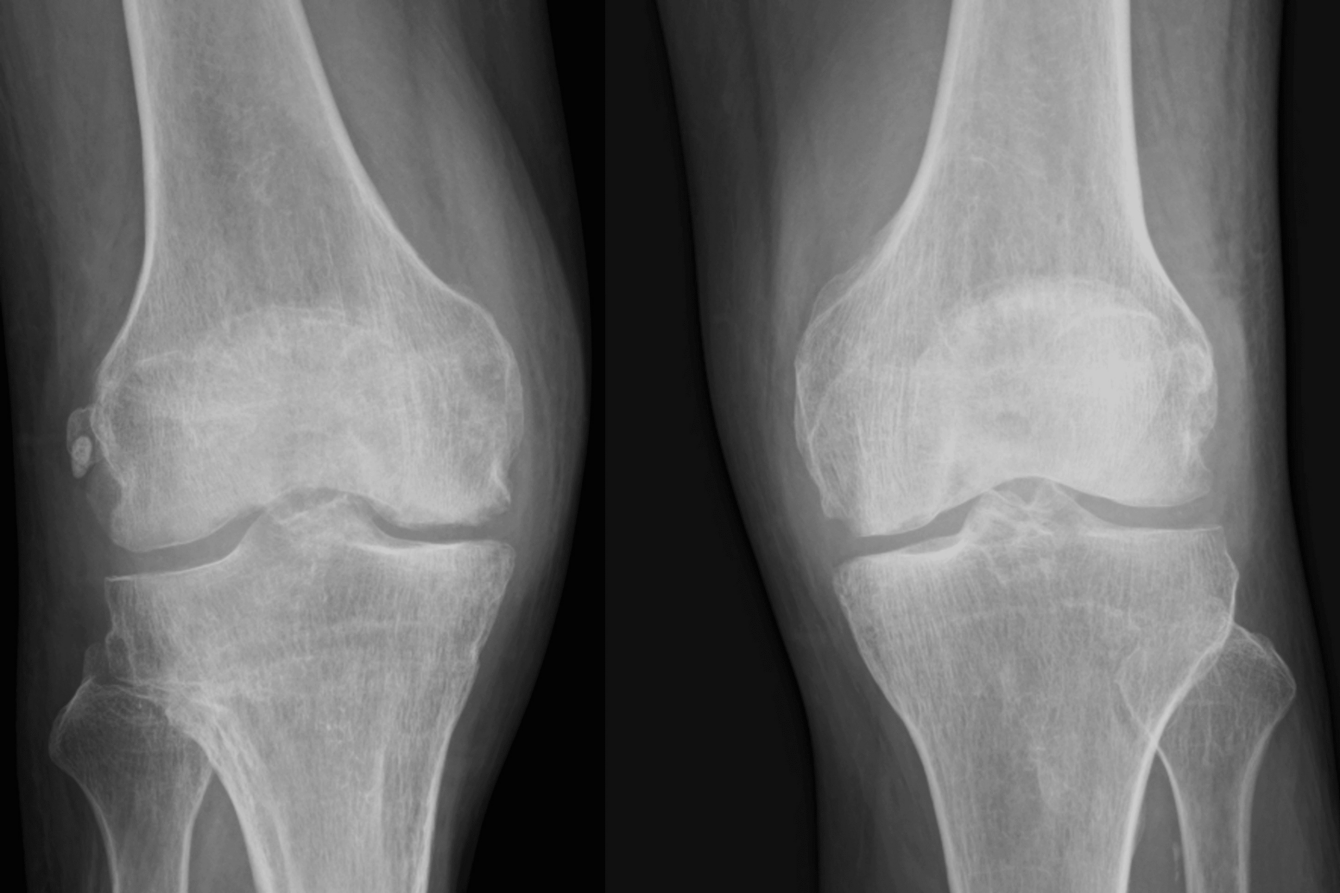
Bilateral knee X-rays after four weeks

Ampicillin was continued for four weeks; during that time, the patient's overall condition improved, joint mobility increased, and inflammatory markers continued to decrease. The serum creatinine returned to 0.7 mg/dL prior to discharge. Plain radiographs of both knees at the time of admission (Figure 5) and four weeks later (Figure 6) showed no evidence of bone destruction.

After completing four weeks of intravenous antibiotic therapy, the patient was transferred to a rehabilitation hospital. At the six-month outpatient follow-up, residual limitations of knee flexion persisted, and the patient required a cane for ambulation. The patient refused further surgical intervention, and follow-up care was discontinued. The isolated GAS was transported to a regional institute of public health and identified as type emm49.

## Discussion

Invasive GAS infections progress rapidly; patients with streptococcal toxic shock syndrome may develop shock within four hours of presentation, and the majority of those with necrotizing fasciitis die within 48 hours of presentation [12]. Therefore, early diagnosis and prompt initiation of treatment are critical for improving outcomes. The rapid diagnosis and initiation of treatment for invasive infections following septic arthritis caused by GAS were facilitated by point-of-care Gram staining of the synovial fluid performed by a physician. Gram staining is a simple, rapid, and cost-effective method that provides immediate results and contributes to the judicious use of broad-spectrum antibiotics. It can detect most bacteria (excluding acid-fast bacilli) and even identify crystals such as monosodium urate and calcium pyrophosphate. However, its limitations include variability depending on the examiner's expertise and significantly reduced sensitivity and specificity after antibiotic administration. Although Gram staining has high specificity (99-100%), its sensitivity (27-81%) is relatively low and variable, particularly for synovial fluid samples [5]. While Gram staining of body fluids is generally more sensitive after centrifugation, one study reported that centrifugation did not improve the sensitivity of Gram staining for synovial fluid [13]. Although the sensitivity and specificity of Gram staining of the synovial fluid are likely to vary depending on the causative organism, we were unable to identify studies that explicitly investigated these differences. Additionally, maintaining a system in which laboratory technicians are always available to perform Gram staining is challenging.

Multiplex polymerase chain reaction (PCR) testing of the synovial fluid has also begun to be utilized, can be performed at any time as long as trained staff and the necessary equipment are available, and may detect pathogens even after antibiotic exposure.; however, its high cost, limited availability for 24-hour operation, longer turnaround time (typically over an hour), limited detection to pathogens included in the assay panel [14], and lack of insurance coverage in Japan restrict its use to a limited number of facilities.

Gram staining and PCR each have distinct strengths and limitations, and a complementary approach that allows for their coexistence would be ideal. However, in real-world clinical settings, especially during nights or weekends when staffing is limited, Gram staining remains a more practical and readily available diagnostic tool.

Point-of-care Gram staining has been performed at the bedside by clinicians in Okinawa, Japan, since 1976, when an infectious disease specialist introduced this method at Okinawa Chubu Hospital [7]. Although this practice has declined in the United States, some institutions in Japan continue to utilize it [6,15]. The clinical application of Gram staining by physicians allows the identification of causative organisms, leading to more accurate diagnoses. Compared to empirical therapy, this approach enables the selection of narrower-spectrum antibiotics, thereby reducing costs and antimicrobial resistance risks [6]. With adequate training, clinicians can proficiently perform Gram staining of high-frequency specimens, such as sputum, urine, and pus, whereas experienced practitioners may achieve higher accuracy using cerebrospinal fluid [10] and synovial fluid Gram stains. While there are variations depending on the facility, Gram staining can generally be performed by first-year residents under the supervision of senior physicians. In institutions with experienced staff, this method can be implemented around the clock. Once residents achieve sufficient proficiency, they may interpret the results and make antibiotic selections independently from their second year onward.

In the present case, purulent arthritis was suspected based on clinical symptoms, and point-of-care Gram staining of the synovial fluid was performed on Sunday morning. Although the bacterial load was low and phagocytosed cocci exhibited Gram uncertainty, extracellular chains of Gram-positive cocci strongly suggested *S. pyogenes* infection. MRSA could not be excluded; therefore, vancomycin was promptly initiated. However, as beta-lactam antibiotics are generally more effective than vancomycin against methicillin-susceptible *Staphylococcus aureus* (MSSA) and streptococci, we also administered ceftriaxone. If point-of-care Gram staining of the synovial fluid had not been performed, initial treatment might have consisted of vancomycin monotherapy to cover common Gram-positive cocci or vancomycin plus ceftriaxone to also cover *N. gonorrhoeae*. However, upon the development of shock, it would have been necessary to broaden coverage further to include Gram-negative organisms such as *P. aeruginosa*, likely prompting the addition of a carbapenem. In contrast, because point-of-care Gram staining suggested *Streptococcus* as the likely pathogen, we were able to target therapy with vancomycin and additionally administer a beta-lactam, which may have enhanced therapeutic efficacy because the blood culture became negative promptly the next day. Gram staining was performed by a physician with the guidance of an infectious disease specialist, and the results were rapidly obtained. The time from synovial fluid aspiration to the availability of Gram staining results was less than 30 minutes, thus enabling the initiation of antibiotic therapy within this short period. We believe this combination contributed to the favorable therapeutic outcome, because invasive GAS infections are known to progress to shock within 4-8 hours even in patients who are not in shock at presentation [16]; in the present case, despite the absence of shock on admission and initiation of treatment within 30 minutes, shock developed 12 hours later, whereas the blood culture had already turned negative by the following day. 

Because of the poor prognosis associated with invasive GAS infections, multidisciplinary management was essential. The patient underwent intermittent aspiration drainage performed by orthopedic surgeons, central venous catheterization and catecholamine administration by intensivists, and continuous renal replacement therapy for renal function preservation. Surgical drainage was performed after the patient's vital signs had stabilized and ultimately resulted in improved survival. However, six months after treatment, the patient exhibited residual knee flexion limitations, thus highlighting the virulence of GAS. Earlier surgical intervention would have been preferable.

Although streptococcal toxic shock syndrome and necrotizing fasciitis can be caused by various emm types, they are most commonly associated with emm1 and emm3 strains [17]. The emm1 genotype of *Streptococcus pyogenes* is a globally widespread and inherently virulent clone. A sublineage known as M1UK, which emerged in England in 2015-2016, produces significantly higher levels of SpeA toxin and has become dominant [18]. Under nutrient-rich conditions, SpeA expression is upregulated approximately 10-fold in M1UK compared to M1global [18]. The prevalence of emm49 GAS is also increasing globally [1,19] and is classified as part of the E3 cluster [20]. The odds ratio for invasive infections is 1.4 (95% CI: 1.3-1.5), indicating a high likelihood of such infections [3]. In this case, although the patient was not immunocompromised, an invasive infection occurred.

## Conclusions

This case demonstrated that point-of-care Gram staining of the synovial fluid facilitated the rapid diagnosis of invasive GAS infection and allowed for the prompt initiation of appropriate therapy without resorting to excessively broad-spectrum antibiotics. Although residual limitation of joint motion persisted, the patient recovered sufficiently to perform daily activities. Point-of-care synovial Gram staining may serve as a useful aid, with 24-hour availability that may vary depending on the institution, for the rapid diagnosis of septic arthritis and for guiding appropriate antimicrobial selection.
